# Efficacy and Safety of Chinese Herbs for the Prevention of the Risk of Renal Damage in Henoch-Schonlein Purpura in Children: Meta-Analysis of Randomized Controlled Trials and GRADE Evaluation

**DOI:** 10.1155/2019/4089184

**Published:** 2019-04-18

**Authors:** Bing Li, Meng Yang, Gai-Li He, Xu-Guang Gao, Le Li, Wen-Sheng Zhai

**Affiliations:** ^1^Henan University of Chinese Medicine, Zhengzhou, Henan 450000, China; ^2^Department of Pediatrics, First Affiliated Hospital, Henan University of Chinese Medicine, Zhengzhou, Henan 450000, China

## Abstract

*Objective. *To evaluate the efficacy and safety of traditional Chinese medicine in preventing kidney damage caused by Henoch-Schonlein Purpura (HSP) in Children by meta-analysis.* Methods. *We systematically searched the main Chinese and English electronic databases and collected randomized controlled trials of Chinese herbs in children with HSP until July 2018. Then we used the bias risk assessment tool in Cochrane Handbook 5. 1. 0 to complete the risk assessment of the included studies. We utilized STATA12.0 and RevMan 5.3 for meta-analysis and GRADE pro. for quality evaluation of evidence.* Result.* (1) Meta-analysis: data from 39 studies, representing 3643 individuals, were included in the analysis. Thirty-seven studies were treated with traditional Chinese medicine for clearing away heat and cooling blood, which were combined. On this basis, subgroup analysis was conducted according to the bias risk of the original study. It showed that Chinese herbs can significantly improve the treatment effect (OR: 4.31, 95% CI [3.34, 5.57],* P* < 0.01) and reduce the risk of renal damage (RR: 0.36; 95% CI [0.21, 0.61],* P* < 0.01) and the risk of recurrence (RR: 0.43, 95% CI [0.34, 0.54],* P*<0.01). (2) Side effect: a total of 7 studies described adverse reactions, and 12 of 319 patients in therapy group had adverse events and 20 of 263 patients in control group. (3) Publication bias: the bias risk Egger's test for the incidence of kidney injury was* P*=0.572, the relapse rate Egger's test was* P*=0.175, the efficiency was combined with the low-risk original study, and the bias risk Egger's test was* P*=0.175. There was not any significant publication bias based on the funnel plot and Egger's test. (4) GRADE evaluation: GRADE evaluation showed that the quality of evidence in the risk of renal damage and recurrence rate was moderate.* Conclusion. *Chinese medicine treatment can prevent the occurrence of renal damage in children with HSP and can reduce the recurrence rate, the incidence of adverse reactions, and the effect in terms of efficiency. However, the quality of the included studies in the meta-analysis and the quality of the evidence of outcomes were not high; the clinical use of the evidence needs to be cautious.

## 1. Introduction

Henoch-Schonlein Purpura (HSP) is a systemic vasculitis syndrome characterized by small vasculitis. It is a common vasculitis in children. HSP usually occurs in children aged 2-6, and the incidence in children is 10.5-20.4/100,000 [1, 2]. Although HSP is a self-limited disease, most of the prognosis is good, but some children can recur, resulting in kidney damage and ultimately poor prognosis. A report by Narchi et al. included 1133 patients in 12 studies and found a 34.2% chance of renal injury. Among these patients with kidney injury, 85% had renal damage within 4 weeks of diagnosis, 91% within 6 weeks, and 97% within 6 months of diagnosis [3]. At present, the main treatment of HSP is antiallergic, analgesic, anticoagulant, and other symptomatic treatments. Glucocorticoids have previously been reported to reduce the risk of kidney injury, but a recent systematic review showed that glucocorticoids can only be used to relieve symptoms of HSP and do not reduce the risk of kidney damage [4].

Many clinical studies reported that Chinese medicine treatment of children with HSP has better curative effect. 6 RCTs were enrolled in 854 patients to observe the intervention effect of TCM syndrome differentiation on the risk of renal injury in HSP patients. The results showed that the risk of kidney damage was reduced to 5.1% in 431 patients treated with traditional Chinese medicine in the intervention group [5–10]. Repeated onset of HSP is an important cause of renal injury. 13 RCT studies reported the impact of TCM treatment on the risk of HSP recurrence. A total of 1260 patients with HSP were treated with TCM. Of 642 patients treated with TCM, 76 had recurrence, while 190 had recurrence in 618 patients in the control group [6, 8, 11–21]. These studies have shown that Chinese medicine therapy has a good therapeutic effect on HSP patients under 18 years old. Zhou et al. included five randomized controlled trials (RCTs) involving 513 children with HSP treated with Salvia miltiorrhiza. The results showed that Salvia miltiorrhiza could reduce the risk of kidney injury in children with HSP [22]. However, the deadline for the systematic evaluation of the literature research is 2012 and only for the study of Salvia miltiorrhiza preparation has not been included in other traditional Chinese medicine syndrome differentiation and treatment related literatures, lack of comprehensive and systematic search. This study will follow the Cochrane system evaluation method to retrieve and evaluate the published RCT of children with HSP treated by traditional Chinese medicine or integrated traditional Chinese and Western medicine. The aim of this meta-analysis of RCTs is to assess the benefits and harms of Chinese medicine used to treat HSP in children and analyze whether it can reduce the risk of renal damage and reduce the recurrence rate.

## 2. Materials and Methods

This meta-analysis was performed according to the PRISMA guidelines [46].

### 2.1. Selection Criteria

#### 2.1.1. Research Objects

The study included patients with the diagnosis of Henoch-Schonlein Purpura who are under 18 years. Gender, race, nationality, and so on were not limited.

#### 2.1.2. Intervention

The experimental group was treated with Chinese medicine or combined with Western medicine basic treatment, and the control group was treated with Western basic medicine. And the follow-up period is not less than two weeks.

#### 2.1.3. Outcomes

The primary outcomes were risk of renal damage and efficient effects. And the secondary outcome measures were recurrence rate and side effects. The included studies should contain at least one of the above outcomes.

#### 2.1.4. Study Design

All included studies were RCTs, and the language was not limited, whether blinding or allocation concealment.

#### 2.1.5. Exclusion Criteria

Studies were excluded if they involved (1) reviews or participants for healthy people; (2) patients with kidney injury; (3) patients with other accompanying diseases; (4) studies with poor quality or obvious errors in data; and (5) no outcomes of interest either reported or cannot calculate or extrapolate based on the available data; (6) for the repeated articles, select the most complete and detailed data into the analysis.

### 2.2. Search Strategy and Methods

We systematically searched Pubmed, Embase, Cochrance online library, CNKI, WanFang Database, CBMdisc, VIP Database, and Clinical Trial Registration Center for reports published before Jun 15, 2018, using a combined text and MeSH heading search strategy with the terms:

“Henoch-Schonlein Purpura”, “anaphylactoid purpura”, “allergic purpura”, “schonlein-henoch purpura”, “henoch schonlein purpura”, “anaphylactic purpura”, “hypersensitivity purpura”, “hypersensitive purpura”, “anapylactoid purpura”, “anapylactoia purpura”, “henoch-schonlein”, “henoch schonlein”, “alllergic purpura”, “hench-schonlein purpura”, “allergy purpura”, “schoenlein-henoch purpura”, “henoch-schoenlein purpura”, “henoch - schonlein purpura”, “‘Purpura, Schoenlein-Henoch'[Mesh]”, “Xijiao Dihuang Decoction”, “Xijiao Dihuang Tang”, “Traditional Chinese Medicine”, “Chinese herb”, “Chinese medicine”, “child”, and “Children”. Reference lists of identified reports for other potentially relevant articles were also checked. Finally, we used Google, Baidu, and other search engines as a supplement to trace the references included in the literature. Take Pubmed as an example; the search strategy is as follows: #1 Search ((“Purpura, Schoenlein-Henoch”[Mesh]) OR Allergic Purpura) OR HSP; #2 Search ((Xijiao Dihuang Decoction) OR Chinese herb) OR Chinese medicine; #3 Search (child) OR children; #1 AND #2 AND #3. Study eligibility was independently determined by two investigators (Bing Li and Le Li); differing decisions were resolved by other members of mutual consensus.

### 2.3. Data Collection and Extraction

Data extraction includes basic information of included studies, such as first author; year of publication; number of experimental and control groups; range of age; observation time; details of interventions; and outcomes; risk of bias. Two trained researchers (Bing Li and Gai-Li He) completed the literature screening and data extraction independently according to the inclusion exclusion criteria and the third party ruled in case of disagreement.

### 2.4. Quality Assessment of the Included Studies

The methodological quality assessment of included studies was independently completed by two investigators (Bing Li and Meng Yang) and any differences were referred to by other members of mutual consensus. We evaluated the risk of bias of included studies using the RCT bias risk assessment tool in Cochrane System Evaluator's Handbook version 5.1.0 [47]. The evaluation was based primarily on seven aspects of selective bias, implementation bias, measurement bias, follow-up bias, reporting bias and other biases, and answered “low risk”, “unclear”, and “high risk” one by one.

### 2.5. Statistical Analysis

We performed statistical analysis using STATA12.0 and RevMan 5.3. The dichotomous variable data used the relative risk (RR) or odds ratio (OR) as the estimate of statistical size, and continuous variable data used mean difference (MD) as the estimate of statistical size and calculated the 95% confidence interval (CI) and the two-side* Z* test* P* value. If the RR did not contain 1, MD did not contain 0, and* P* <0.05 was considered statistically significant [48]. The Q statistic test and I^2^ statistic were used to estimate the percentage of variability across studies due to between-study heterogeneity. If I^2^<50% and* P*>0.1, the fixed-effects model was chosen. Otherwise, we generated pooled estimates across studies using random-effects meta-analysis. Besides, each individual trial was removed for carrying out a sensitivity analysis in the meta-analysis [48, 49]. The publication bias for risk of renal damage, recurrence rate, and efficiency was statistically evaluated using Egger's and Begg's tests [50], and* P* values less than 0.05 were considered to have a significant heterogeneity. If there was bias, we use clipping to estimate the exact number of deletion studies [23]. The incidence of adverse reactions was compared by chi-square test and realized by STATA12.0.

### 2.6. Evidence Quality Evaluation

The results of the meta-analysis were evaluated using the GRADE method and whether degradation was considered in terms of risk of bias, inconsistency, indirectness, accuracy, and publication bias, divided into “high quality”, “moderate quality”, “low quality”, and “very low quality” [11].

## 3. Results

### 3.1. Literature Screening Results

**Figure 1:**
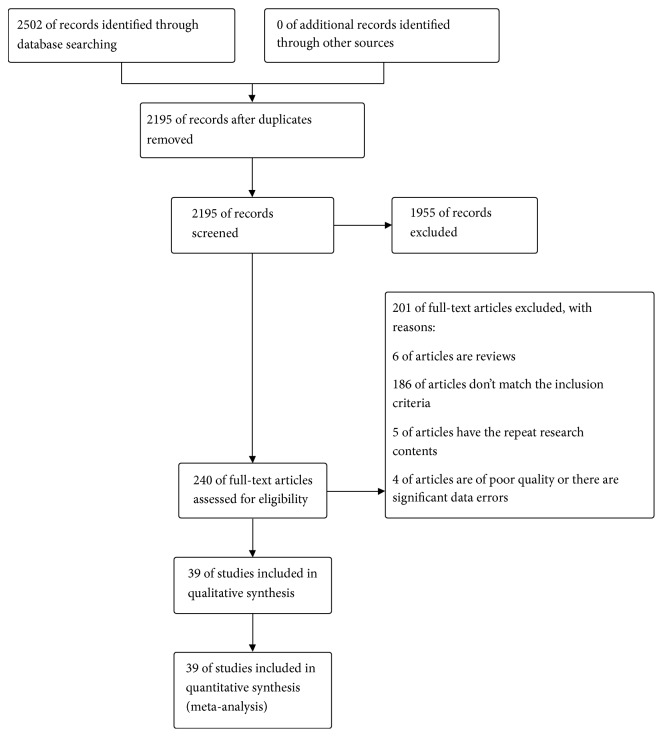
Study selection flow chart.

During initial electronic search, 2502 articles were identified, of which 307 were excluded including duplicates and irrelevant articles. The full texts of the selected 240 trials were retrieved, and after detailed evaluation, 39 RCTs that included 3643 patients were eventually included for meta-analysis. No additional trials were identified in manual search.  Figure 1 is the flow chart of study selection process.

### 3.2. Basic Characteristics and Risk of Bias of Included Studies

The 39 RCTs included were all Chinese, with a total of 3643 cases of HSP [5–10, 12–21, 23–45]. The follow-up duration of included trials ranged from 2 weeks to 1 year. Baseline conditions were described in all 39 studies, and the two groups were comparable in terms of age, gender, duration of disease, and severity of the disease or were described as not statistically significant (t test* P*>0.05). The bias risk assessment results of 39 studies were 17 low risk, 16 unclear, and 6 high risk. The baseline characteristics and the risk of bias of HSP patients (n=3643) were presented in Table 1.

### 3.3. Risk Bias Assessment of Included Studies

**Figure 2:**
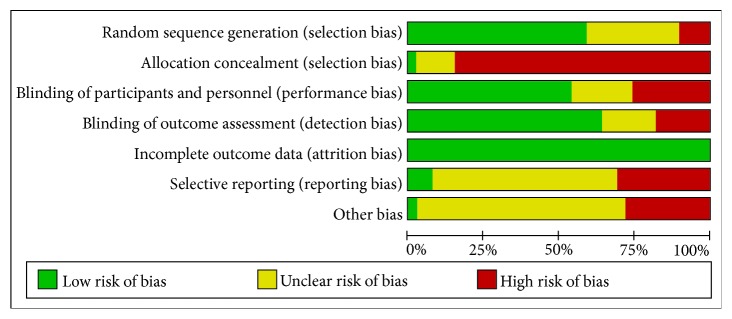
Risk bias assessment for included studies.

Of the 39 eligible studies, all of the trials described randomization. 10 of the studies were reported as random number tables [9, 13, 16, 18, 19, 21, 24, 28, 29, 31, 33, 37, 42, 45], the other studies did not describe accurate random sequence generation methods, and none of the included trials were lost to follow-up.  Figure 2 showed the risk bias assessment for included studies.

### 3.4. Results of Meta-Analysis

Because the quality of the original study was not equal, the subgroup analysis was carried out according to the bias risk of the inclusion study in the meta-analysis. Because the interventions adopted in gui2017 and ma2018 are not homogeneous with 37 other articles, the analysis is listed separately and not included.

#### 3.4.1. Risk of Renal Damage

**Figure 3:**
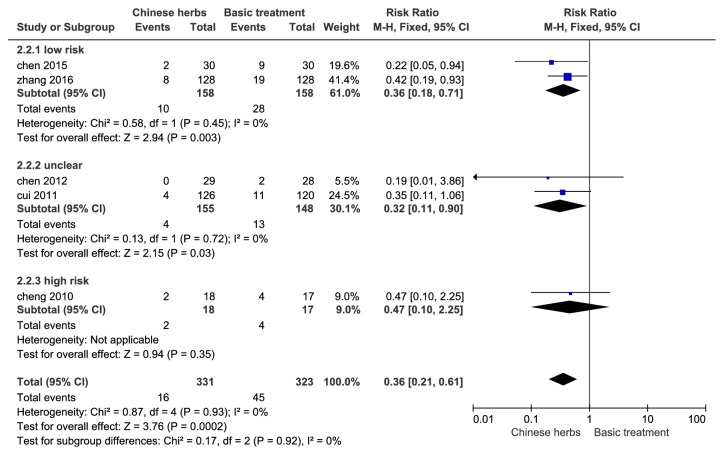
Forest plot of renal injury rate of HSP in children (RR, relative risk; CI, confidence interval).

Data from a total of 654 HSP patients included in 5 studies were used to evaluate the effect of Chinese herbs of clearing heat and cooling blood on renal injury rate. Heterogeneity test* P* = 0.93, I^2^ = 0.0%, and this result indicated no significant heterogeneity between studies, so we chose a fixed effect model calculations; the end result showed that Chinese herbs can significantly reduce the risk of renal injury (RR: 0.36; 95% CI [0.21, 0.61],* P *< 0.01). Subgroup analysis showed that the combined effects of low-risk studies were (RR: 0.36; 95% CI [0.18, 0.71], P < 0.01), which was consistent with the total effects; see Figure 3.

#### 3.4.2. Efficient

**Figure 4:**
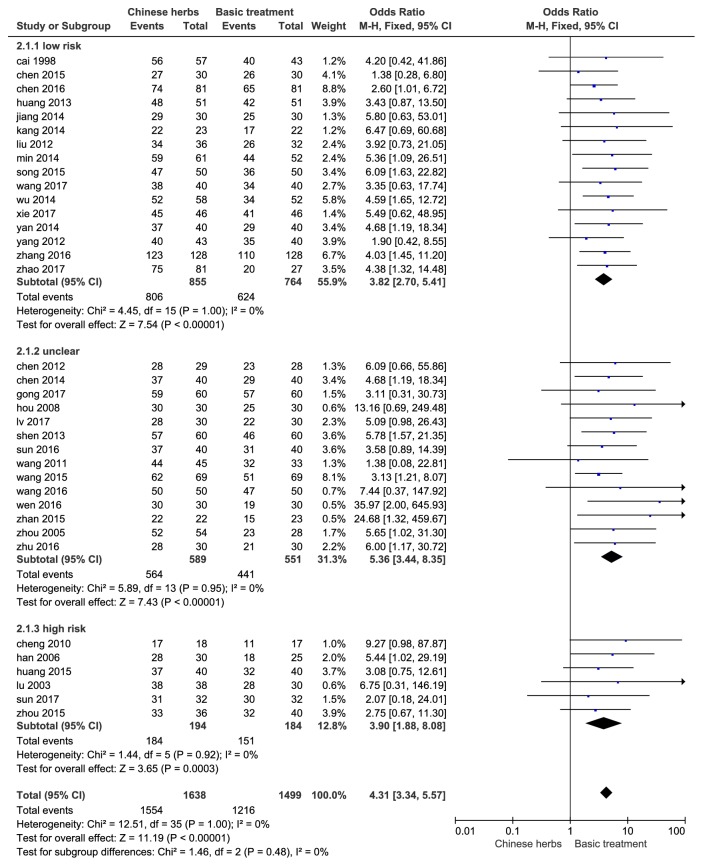
Forest plot of efficiency of HSP in children (OR, relative risk; CI, confidence interval).

A total of 3137 patients were enrolled in 36 studies to evaluate the effect of treatment in the two groups. Heterogeneity test* P*=1, I^2^=0.0%, this result showed no significant heterogeneity between studies, and then we used a fixed effect model calculation; eventually it indicated that Chinese herbs can significantly improve efficacy (OR: 4.31, 95% CI [3.34, 5.57],* P* < 0.01). Subgroup analysis showed that the combined effects of low-risk studies were OR: 3.82, 95% CI [2.70, 5.41],* P* < 0.01, which was consistent with the total effects; see Figure 4.

#### 3.4.3. Recurrence Rate

**Figure 5:**
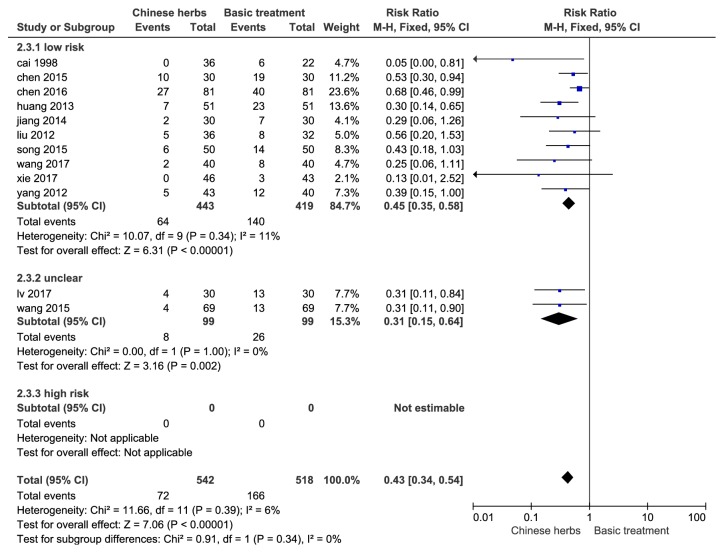
Forest plot of recurrence rate (RR, relative risk; CI, confidence interval).

12 studies included 1060 patients who reported changes in the two groups. Heterogeneity test* P*=0.39, I^2^=6%, and the fixed effect model meta-analysis showed statistically significant difference (RR: 0.43, 95% CI [0.34, 0.54],* P*<0.01). Subgroup analysis showed that the combined effects of low-risk studies were RR: 0.45; 95% CI [0.35, 0.58],* P *< 0.01, which was consistent with the total effects; see Figure 5.

#### 3.4.4. Side Effects

7 studies including 582 patients described the risk of adverse effects. Among them, 12 of the 319 patients in the treatment group had adverse reactions; the risk of adverse reactions was 3.8%. In the control group, 263 cases had adverse reactions in 20 cases; the risk of side effect was 7.6%. Chi-square test was used to compare the incidence of adverse reactions between the two groups. The results showed that the chi-square value was 4.097; the* P* value was 0.043, < 0.05, suggesting that there was a significant difference between the two groups.

#### 3.4.5. Results of Studies on Chinese Patent Medicines as Interventions

Gui2017 included 200 cases, and the effective combined effects were OR: 5.79, 95% CI [2.27, 14.77], the combined effects of kidney damage were RR: 0.24, 95% CI [0.10, 0.56], and the combined effects of recurrence were RR: 0.13, 95% CI [0.04, 0.40]. Sixty cases were included in Ma2018. The effective combined effect was OR: 16.18, 95% CI [0.87, 301.62]. These results are consistent with the above combined results, indicating that TCM treatment can indeed improve the efficiency, reduce the risk of kidney and kidney damage, and reduce the recurrence rate.

### 3.5. Publication Bias Assessment

**Figure 6:**
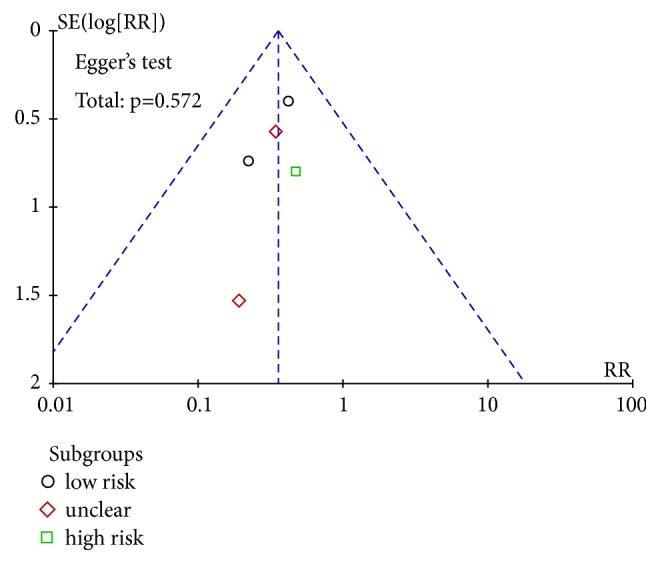
Funnel plot of renal injury rate.

**Figure 7:**
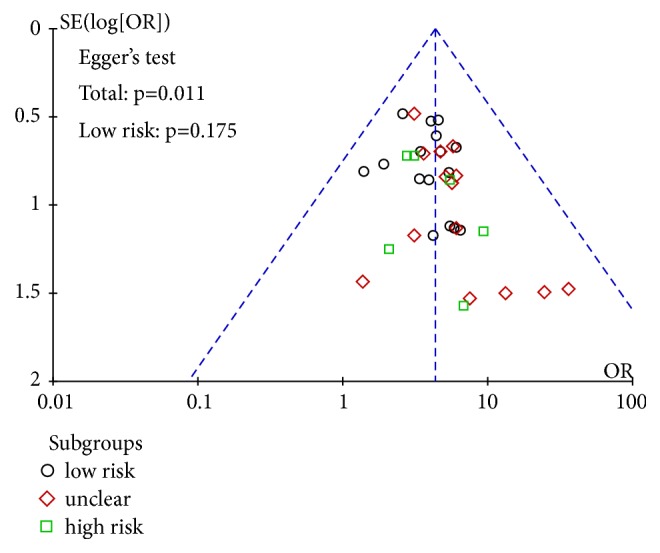
Funnel plot of efficiency.

**Figure 8:**
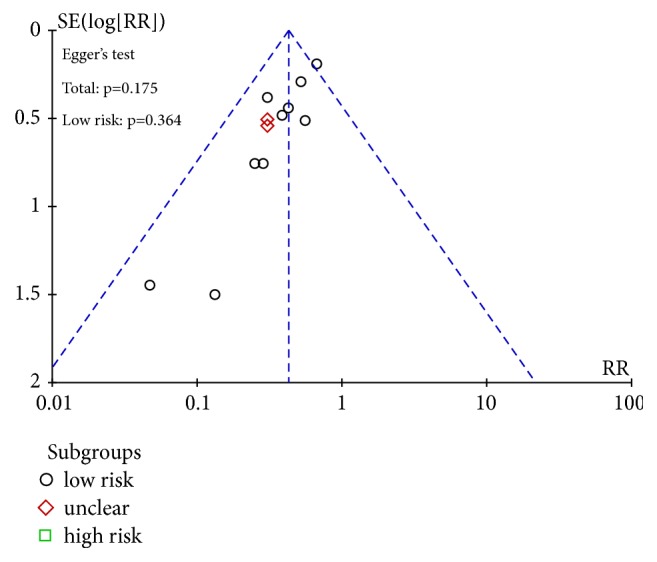
Funnel plot of recurrence rate.

Publication bias of renal injury rate was detected, funnel plot showed no significant bias, Egger's test* P* = 0.572; see Figure 6. Egger's test was used to test the efficiency of publication bias. 37 studies were combined and the result was* P*=0.011, suggesting that publication bias existed. Further clipping method was used to test the size of publication bias. It was concluded that 7 studies needed to be added to obtain more stable results, indicating that publication bias was larger. 16 of the studies were of high quality, which were combined and tested by Egger's test. The results showed that* P* = 0.175 > 0.05, suggesting that there was no publication bias; see Figure 7. In summary, the efficient meta-analysis results are more robust. The recurrence rate was detected by publication bias, and there was no obvious bias in funnel graph. 12 studies were combined for Egger's test and* P*=0.175 was obtained, suggesting that there was no publication bias; see Figure 8.

### 3.6. Evidence Quality Evaluation

We used the GRADE pro system to evaluate the quality of evidence for the primary outcomes: renal injury rate, recurrence rate, and efficient. The RCT was preset to the highest level of evidence in the GRADE evidence quality assessment and was processed according to five degradation factors. The results suggested that the quality of the evidence in the renal injury rate and recurrence rate and efficiency were intermediate. See Table 2.

## 4. Discussion

This meta-analysis included 39 RCTs with a total of 3643 HSP cases. Among them, the intervention measures were mainly traditional Chinese medicine (TCM) syndrome differentiation, TCM self-preparation, Xijiao Dihuang Decoction, and Chinese patent medicines. The shortest treatment or follow-up time was 2 weeks and the longest was 1 year. The results of meta-analysis showed that Chinese medicine treatment of children with HSP was better and safer. There were 6 studies in 854 cases describing the prevention of renal injury. A total of 1260 cases in 13 studies described the impact on recurrence rate. In 7 studies, 582 cases described adverse reactions. Meta-analysis showed Chinese medicine has a good effect.

The results of this study were consistent with previous studies. The system evaluation results of Yan Li and Hua-Chun Chen are also consistent with the results of this study [51, 52]. Chang-Jiang Zhao et al.'s research shows that Xijiao Dihuang Decoction can shorten the time of symptom disappearance in children with HSP and improve the curative effect [31]. Xiu- Li Cui's study found that the incidence of kidney injury was statistically significant in children treated with the main prescription of Xijiao Dihuang Decoction compared with conventional basic treatment [25].

A number of studies have found that traditional Chinese medicine or integrated traditional Chinese and Western medicine treatment of children with HSP is better than Western medicine, but there is less research on whether Chinese medicine can reduce the risk of kidney damage, and the sample size is less. In this analysis, a total of 39 RCTs were included through a systematic database search to compare the effectiveness of TCM and Western medicine in the treatment of HSP in children and to reduce the risk of kidney injury, recurrence rate and adverse reactions. Our study found that traditional Chinese medicine or combination of traditional Chinese and Western medicine has statistically significant differences in improving efficacy, reducing the risk of kidney damage, and reducing the rate of recurrence and the incidence of adverse reactions. The funnel plots for each outcome showed no publication bias in this meta-analysis.

This meta-analysis mainly has the following limitations: (1) Small sample size: although a comprehensive search has been conducted, there are fewer studies that meet the inclusion criteria, and there are few reports on the incidence of kidney damage and recurrence. It is necessary to increase the sample size in future research. (2) The quality of the original research included is not high: the risk bias assessment results of the included studies were poor and the quality was moderate, which may affect the reliability of the conclusions of this meta-analysis. In future research, the quality of clinical research needs to be further improved, which provides a more reliable basis for the efficacy of Chinese medicine in the treatment of HSP. (3) The follow-up time of the included studies was inconsistent and many studies did not mention follow-up, which made it difficult to research the incidence of kidney damage and had an impact on the reliability of the conclusions of this analysis.

We evaluated the quality of GRADE evidence for the two outcomes of the risk of renal injury and recurrence rate. The results showed that the two outcomes of kidney damage rate and recurrence rate were medium quality. Through the analysis of the included studies, the main reason for this result is that the study design is not rigorous and the sample size is insufficient. Based on the current research results, this meta-analysis shows that Chinese medicine treatment of children with HSP is effective, and it can reduce the risk of kidney damage and reduce the recurrence rate. However, due to the low quality of GRADE evaluation of primary outcomes, based on the limitations of existing research, this meta-analysis conclusion still needs to be confirmed by large-sample, multicenter, high-quality RCT studies.

## 5. Conclusions

Chinese medicine can reduce the risk of kidney injury and recurrence rate of children with HSP. However, the quality level of evidence is not high and requires multicenter, large-sample randomized controlled trials.

## Figures and Tables

**Table 1:** Characteristics of randomized controlled trials of Chinese herbs for HSP in children.

Study ID	Age	Sample size	Experimental	Control group	Observation	Outcomes	Risk of bias
	(year)	(Experimental/Control)	group measures	measures	time		(Low/Unclear/High)
Cai 1998 [23]	1-18	57/43	The experimental group used Xijiao Dihuang Decoction, clinical routine dose, 2 times a day	The control group received Basic western medicine treatment	6 months	(1)(3)	Low

Chen 2014 [24]	3-12	40/40	Xijiao Dihuang Decoction, clinical routine dose, 2 times a day. The basic treatment is same as the control group	The control group received Basic western medicine treatment	2 weeks	(1)(4)	Unclear

Cui 2011 [25]	4-13	126/120	The experimental group used Xijiao Dihuang Decoction, clinical routine dose, 2 times a day	The control group received Basic western medicine treatment	6 months	(2)	Unclear

Han 2006 [26]	6-14	30/25	Xijiao Dihuang Decoction, clinical routine dose, 2 times a day. The basic treatment is same as the control group	The control group received Basic western medicine treatment	2 weeks	(1)	High

Lu 2003 [5]	4-14	38/30	Xijiao Dihuang Decoction, clinical routine dose, 2 times a day. The basic treatment is same as the control group	The control group received Basic western medicine treatment	2 weeks	(1)	High

Sun 2016 [27]	3-14	40/40	The experimental group used Xijiao Dihuang Decoction, clinical routine dose, 2 times a day	The control group received Basic western medicine treatment	2 weeks	(1)	Unclear

Wu 2014 [12]	3-16	58/52	Xijiao Dihuang Decoction, clinical routine dose, 2 times a day. The basic treatment is same as the control group	The control group received Basic western medicine treatment	2 weeks	(1)	Low

Yan 2014 [28]	3-12	40/40	Xijiao Dihuang Decoction, clinical routine dose, 2 times a day. The basic treatment is same as the control group	The control group received Basic western medicine treatment	2 weeks	(1)(4)	Low

Yang 2012 [29]	3-16	43/40	The experimental group used Xijiao Dihuang Decoction, clinical routine dose, 2 times a day	The control group received Basic western medicine treatment	3 weeks	(1)(3)	Low

Zhan 2015 [30]	1-18	22/23	The experimental group used Xijiao Dihuang Decoction, clinical routine dose, 2 times a day	The control group received Basic western medicine treatment	6 weeks	(1)	Unclear
Zhao 2017 [31]	2-14	81/27	Xijiao Dihuang Decoction, clinical routine dose, 2 times a day. The basic treatment is same as the control group	The control group received Basic western medicine treatment	2 weeks	(1)(4)	Low

Wang 2016 [6]	5-14	50/50	Modified Huaban Decoction, clinical routine dose, 2 times a day. This Formula consists of Chinese herbs for cooling blood and eliminating spots and removing extravasated blood and dredge Collaterals. The basic treatment is same as the control group	The control group received Basic western medicine treatment	1 year	(1)	Unclear

Chen 2015 [7]	3-10	30/30	Wuteng Granules, clinical routine dose, 2 times a day. This Granule consists of Chinese herbs for cooling blood and eliminating wind and removing extravasated blood and dredge Collaterals. The basic treatment is same as the control group	The control group received Basic western medicine treatment	8 weeks	(1)(2)(3)(4)	Low

Chen 2012 [32]	4-11	29/28	Xijiao Dihuang Decoction, clinical routine dose, 2 times a day. The basic treatment is same as the control group	The control group received Basic western medicine treatment	3 weeks	(1)(2)(4)	Unclear

Ma 2018 [13]	5-18	30/30	Kunxian Granules, clinical routine dose, 2 times a day. The basic treatment is same as the control group	The control group received Basic western medicine treatment	2 months	(1)	Unclear

Lv 2017 [8]	6-15	30/30	Liangxue Xiaodian Decoction, clinical routine dose, 2 times a day. The composition of this Formula is based on the addition and subtraction of Xijiao Dihuang Decoction. The basic treatment is same as the control group	The control group received Basic western medicine treatment	4 weeks	(1)(3)	Unclear

Gui 2017 [33]	3-14	100/100	Huaiqihuang Granules, clinical routine dose, 2 times a day. The basic treatment is same as the control group	The control group received Basic western medicine treatment	3 months	(1)(2)(3)	Low

Shen 2013 [14]	4-15	60/60	Shitan Decoction, clinical routine dose, 2 times a day. This Formula consists of Chinese herbs for cooling blood and clearing away heat and detoxification. The basic treatment is same as the control group	The control group received Basic western medicine treatment	2 weeks	(1)	Unclear

Wang 2017 [34]	3-17	40/40	Self-Designed Formula, clinical routine dose, 2 times a day. This Formula consists of Chinese herbs for cooling blood and eliminating spots and clearing away heat and detoxification. The basic treatment is same as the control group	The control group received Basic western medicine treatment	2 weeks	(1)(3)	Low

Hou 2008 [35]	12-18	30/30	Xiaofeng Formula, clinical routine dose, 2 times a day. This Formula consists of Chinese herbs for cooling blood and eliminating wind and removing extravasated blood and dredge Collaterals. The basic treatment is same as the control group	The control group received Basic western medicine treatment	2 weeks	(1)	Unclear

Gong 2017 [15]	3-16	60/60	Zidian Zhenxiao Granules, clinical routine dose, 2 times a day. This Formula consists of Chinese herbs for cooling blood and clearing away heat and removing extravasated blood and dredge Collaterals. The basic treatment is same as the control group	The control group received Basic western medicine treatment	2 weeks	(1)	Unclear

Song 2015 [16]	5-13	30/30	Yinqiao Formula, clinical routine dose, 2 times a day. This Formula consists of Chinese herbs for cooling blood and clearing away heat. The basic treatment is same as the control group	The control group received Basic western medicine treatment	6 months	(1)(3)	Low

Huang 2013 [36]	1-12	51/51	Self-Designed Formula, clinical routine dose, 2 times a day. The composition of this Formula is based on the addition and subtraction of Xijiao Dihuang Decoction. The basic treatment is same as the control group	The control group received Basic western medicine treatment	6 months	(1)(3)	Low

Kang 2014 [17]	2-13	23/22	Choose the appropriate treatment according to different syndrome types. The basic treatment is same as the control group	The control group received Basic western medicine treatment	3 weeks	(1)	Low

Wang 2015 [9]	5-17	69/69	Self-Designed Formula, clinical routine dose, 2 times a day. This Formula consists of Chinese herbs for cooling blood and eliminating wind and removing extravasated blood and dredge Collaterals. The basic treatment is same as the control group	The control group received Basic western medicine treatment	2 weeks	(1)(3)	Unclear

Cheng 2010 [18]	6-14	18/17	Choose the appropriate treatment according to different syndrome types. The basic treatment is same as the control group	The control group received Basic western medicine treatment	2 weeks	(1)(2)(4)	High

Chen 2016 [37]	3-14	81/81	Self-Designed Formula, clinical routine dose, 2 times a day. The composition of this Formula is based on the addition and subtraction of Xijiao Dihuang Decoction. The basic treatment is same as the control group	The control group received Basic western medicine treatment	2 weeks	(1)(3)(4)	Low

Sun 2017 [38]	3-12	32/32	Salvia Injection, 10mL a day. The basic treatment is same as the control group	The control group received Basic western medicine treatment	2 weeks	(1)	High

Zhu 2016 [19]	0.5-6	30/30	Self-Designed Formula, clinical routine dose, 2 times a day. The composition of this Formula is based on the addition and subtraction of Xijiao Dihuang Decoction. The basic treatment is same as the control group	The control group received Basic western medicine treatment	2 weeks	(1)	Unclear

Liu 2012 [20]	3-14	36/32	Choose the appropriate treatment according to different syndrome types. The basic treatment is same as the control group	The control group received Basic western medicine treatment	4 weeks	(1)(3)	Low

Xie 2017 [39]	1-12	46/46	Modified Huaban Decoction, clinical routine dose, 2 times a day. This Formula consists of Chinese herbs for cooling blood and eliminating spots and removing extravasated blood and dredge Collaterals. The basic treatment is same as the control group	The control group received Basic western medicine treatment	4 weeks	(1)(3)	Low

Zhou 2015 [40]	4-16	40/40	Choose the appropriate treatment according to different syndrome types. The basic treatment is same as the control group	The control group received Basic western medicine treatment	2 weeks	(1)	High

Min 2014 [41]	1-17	61/52	Self-Designed Formula, clinical routine dose, 2 times a day. The composition of this Formula is based on the addition and subtraction of Xijiao Dihuang Decoction. The basic treatment is same as the control group	The control group received Basic western medicine treatment	3 weeks	(1)	Low

Huang 2015 [42]	2-8	40/40	Choose the appropriate treatment according to different syndrome types. The basic treatment is same as the control group	The control group received Basic western medicine treatment	2 weeks	(1)	High

Wang 2011 [43]	2-13	45/33	Kangmin Xiaodian Decoction, clinical routine dose, 2 times a day. This Formula consists of Chinese herbs for cooling blood and eliminating wind and removing extravasated blood and dredge Collaterals. The basic treatment is same as the control group	The control group received Basic western medicine treatment	3 weeks	(1)	Unclear

Wen 2016 [21]	3-14	30/30	Liangxue Xiaoban Decoction, clinical routine dose, 2 times a day. This Formula consists of Chinese herbs for cooling blood and eliminating spots. The basic treatment is same as the control group	The control group received Basic western medicine treatment	2 weeks	(1)	Unclear

Jiang 2014 [44]	4-11	30/30	Self-Designed Formula, clinical routine dose, 2 times a day. This Formula consists of Chinese herbs for cooling blood and eliminating spots and clearing away heat. The basic treatment is same as the control group	The control group received Basic western medicine treatment	2 weeks	(1)(3)	Low

Zhou 2005 [10]	2-14	54/28	Choose the appropriate treatment according to different syndrome types	The control group received Basic western medicine treatment	2 weeks	(1)	Unclear

Zhang 2016 [45]	6-16	128/128	Zidan Particles, clinical routine dose, 2 times a day. This Particles consists of Chinese herbs for cooling blood and clearing away heat and removing extravasated blood and dredge Collaterals. The basic treatment is same as the control group	The control group received Basic western medicine treatment	4 weeks	(1)(2)	Low

(1) efficient; (2) risk of renal damage; (3) recurrence rate; (4) side effect.

**Table 2:** Evaluation of GRADE in children with HSP treated by Chinese herbs.

Chinese herbs compared to Basic treatment for HSP						
Patient or population: patients with HSP						
Settings: RCT						
Intervention: Chinese herbs						

Outcomes	Illustrative comparative risks^**∗**^		Relative effect (95% CI)	No of Participants (studies)	Quality of the evidence (GRADE)	Comments
	(95% CI)					
	Assumed risk	Corresponding risk				
	Basic treatment	Chinese herbs				

efficient	Study population		OR 4.31 (3.34 to 5.57)	3137 (36 studies)	⊕⊕⊕ ⊝ moderate^1^	
	811 per 1000	949 per 100				
		(935 to 960)				
	Moderate					
	808 per 1000	948 per 1000				
		(934 to 959)				

renal injury rate	Study population		RR 0.36 (0.21 to 0.61)	654 (5 studies)	⊕⊕⊕ ⊝ moderate^1^	
	139 per 1000	50 per 1000				
		(29 to 85)				
	Moderate					
	148 per 1000	53 per 1000				
		(31 to 90)				

recurrence rate	Study population		RR 0.43 (0.34 to 0.54)	1060 (12 studies)	⊕⊕⊕ ⊝ moderate^1^	
	320 per 1000	138 per 1000				
		(109 to 173)				
	Moderate					
	276 per 1000	119 per 1000				
		(94 to 149)				

^*∗*^The basis for the *assumed risk* (e.g., the median control group risk across studies) is provided in footnotes. The *corresponding risk* (and its 95% confidence interval) is based on the assumed risk in the comparison group and the *relative effect* of the intervention (and its 95% CI).

*CI*: confidence interval; *RR*: risk ratio; *OR*: odds ratio.

GRADE Working Group grades of evidence.

*High quality*: further research is very unlikely to change our confidence in the estimate of effect.

*Moderate quality*: further research is likely to have an important impact on our confidence in the estimate of effect and may change the estimate.

*Low quality*: further research is very likely to have an important impact on our confidence in the estimate of effect and is likely to change the estimate.

*Very low quality*: we are very uncertain about the estimate.

^1^ Random, blinded risk of bias.

## Data Availability

The data included original studies and meta-analysis file with suffix.rm5. The data used to support the findings of this study are available from the corresponding author upon request.
